# Safety and efficacy of esketamine for postoperative analgesia in pediatric patients with hypospadias

**DOI:** 10.3389/fsurg.2023.1131137

**Published:** 2023-04-04

**Authors:** Yong Xu, Quan Chen, Ping Li, Xingrong Song

**Affiliations:** ^1^ Department of Anesthesiology, The First School of Clinical Medicine of Jinan University, Guangzhou, China; ^2^ Department of Anesthesiology, The First Affiliated Hospital of Jinzhou Medical University, Jinzhou, China; ^3^ Department of Anesthesiology, Guangzhou Women and Children’s Medical Center, Guangzhou, China

**Keywords:** esketamine, postoperative analgesia, pediatric, hypospadias, opioids

## Abstract

**Objective:**

To explore the safety and efficacy of the combination of continuous intravenous infusion of esketamine with sacral block for postoperative analgesia in pediatric patients undergoing surgery for hypospadias

**Methods:**

Pediatric patients (*n *= 77) undergoing surgery for hypospadias were randomized into two groups: a hydromorphone group (H group, initial dose, 0.02 mg/kg; maintenance dose, 0.01 mg/kg/h) or an esketamine group (E group, initial dose, 0.3 mg/kg; maintenance dose 0.15 mg/kg/h). Caudal epidural block involved injection of 0.2% ropivacaine 1 ml/kg through the sacral hiatus. Age, weight, grade of hypospadias, intraoperative blood loss, operative time, and awaking time of patients were recorded. The Face, Legs, Activity, Cry and Consolability (FLACC) scale and Ramsay sedation scores were recorded when leaving the PACU (0 h) and at postoperative 2, 6, 12, 24, 36, and 48 h. The Pediatric Anesthesia Emergence Delirium Scale (PAED), incidence of hypotension, respiratory depression, nausea and vomiting and pruritus, and the time to first bowel movement after surgery were recorded.

**Results:**

There were no significant differences in demographic and clinical characteristics between the H group and the E group. There were no significant differences in FLACC scores at postoperative 0, 2, 6, 12, 24, 36, and 48 h in intra-group and inter-group comparisons. There were no significant differences in Ramsay sedation scores at postoperative 0, 2, 6, 12, 24, 36, and 48 h in the intra-group comparisons. Ramsay sedation scores were significantly lower at postoperative 2, 12, and 36 h in the H group compared to the E group. There were no significant differences in the PAED scale or incidence of nausea and vomiting or pruritus between the H group and the E group. The incidence of hypotension and respiratory depression was significantly lower, and the time to first bowel movement was significantly shorter in the E group compared to the H group. Urinary tryptophan, 5-hydroxytryptamine and substance P levels were significantly lower but arginine was significantly higher in the E group compared to the H group.

**Conclusions:**

The combination of continuous intravenous infusion of esketamine with sacral block provided safe and effective postoperative analgesia for pediatric patients undergoing surgery for hypospadias.

**Trial registration:**

Chinese Clinical Trial Register ChiCTR2200066967. Clinical trial registry URL: http://www.chictr.org.cn/edit.aspx?pid=185042&htm=4

## Introduction

Persistent postoperative pain in pediatric patients has been associated with new onset behavioral problems such as nightmares and separation anxiety, and changes in sensitivity, including pain and anxiety, to later medical procedures (1). Underlying mechanisms may result in pain hypersensitivity due to activation of the nociceptive system and plasticity of pain neuropathways (2). There remains an unmet clinical need for effective postoperative analgesia in pediatric patients. Evidence suggests 40% of pediatric patients experience moderate to severe pain after surgery, and 75% receive insufficient postoperative analgesia (3). Safe and effective postoperative analgesia in pediatric patients can reduce morbidity, promote successful recovery and rehabilitation, increase patient and parent satisfaction, and reduce the burden on healthcare services (4). Hypospadias is a common congenital malformation of the urinary tract. Surgical correction is usually performed early in life. Postoperative pain can be severe, as surgical repair involves a large wound surface with an extensive nerve distribution (5). Postoperative pain control should be optimized in this patient population.

Analgesic drugs must be effective and safe. Opioids are commonly used, but have been associated with dangerous adverse effects in pediatric patients, such as respiratory depression. Esketamine is the active enantiomer of ketamine, and has more potent pharmacological effects than racemic ketamine. The anxiolytic, sedative, and hypnotic effects of esketamine make it an ideal postoperative analgesic for pediatric patients undergoing surgery. In pediatric patients undergoing hydrostatic or pneumatic reduction of intussusceptions, procedural sedation with esketamine was associated with a higher success rate, lower recurrence rate, shorter duration, and shorter length of hospital stay compared to morphine analgesia (6). In pediatric patients undergoing reduction of forearm fractures, procedural sedation with esketamine achieved acceptable outcomes (7). Currently, there is a paucity of data supporting the use of esketamine in pediatric patients undergoing surgery for hypospadias, and clinical studies are required to gather empirical evidence.

This prospective, randomized, controlled, and double-blind clinical trial explored the safety and efficacy of the combination of continuous intravenous infusion of esketamine with sacral block for postoperative analgesia in pediatric patients undergoing surgery for hypospadias.

## Materials and methods

### Study population

The study protocol was approved by the Guangzhou Women and Children Medical Center institutional review board [Protocol No. (2020)26001] and registered at ClinicalTrials.gov (registration No. ChiCTR2200066967, http://www.chictr.org.cn/edit.aspx?pid=185042&htm=4). Pediatric patients undergoing surgery for hypospadias at the Guangzhou Women and Children Medical Center between November 1, 2021 and April 30, 2022 were eligible for this study. Inclusion criteria were: (1) age 3 months to 6 years; (2) American Society of Anesthesiologists (ASA) physical status I and II; and (3) scheduled to undergo surgery under general anesthesia. Exclusion criteria were: (1) contraindications to sacral block, including sacral malformation and abnormal coagulation function; (2) preoperative signs of difficult airway; (3) enlarged tonsils, adenoidal hypertrophy or arrhythmia; (4) hepatitis and/or renal dysfunction; (5) increased intracranial pressure and/or intracerebral hemorrhage; (6) pheochromocytoma and/or primary hyperaldosteronism; (7) severe cardiovascular disease; (8) glaucoma; (9) hyperthyroidism; (10) mental and neurological disease; (11) history of complications in post-anesthesia recovery and/or to analgesic use; or (12) allergic reactions to esketamine, opioids or their components.

Included patients were randomized into two groups using a random number table: patients receiving hydromorphone (H group) or patients receiving esketamine (E group).

### Surgical procedure

Patients were required to fast before surgery. Perioperative monitoring included electrocardiogram, heart rate (HR), non-invasive blood pressure (BP) measurements, pulse oximetry, and end-tidal CO_2_ levels. Sodium bicarbonate Ringer's solution was administered by intravenous infusion (4 ml/kg/h). Anesthesia was induced by bolus injection of cisatracurium 0.2 mg/kg, sufentanil 0.3 µg/kg, and propofol 2 mg/kg. Endotracheal or laryngeal mask ventilation was performed after 3 min, with subsequent mechanical ventilation. Caudal epidural block involving injection of 0.2% ropivacaine 1 ml/kg through the sacral hiatus was performed five minutes before skin sterilization, with the patient in the lateral lying position. Anesthesia was maintained with 2%–3% sevoflurane. Intraoperative HR and BP targets were maintained within 20% of their respective baseline values using vasoactive drugs. Intravenous infusion pumps for analgesia were started 10 min before the end of surgery. After the procedure, sevoflurane was discontinued, and patients were transferred to the postanesthesia care unit (PACU). Tracheal extubation was performed when the patient regained consciousness and spontaneous breathing. Patients remained in the PACU until their vital signs (HR, BP, SpO_2_) remained stable for at least 20 min.

### Postoperative analgesia

Ten minutes before the surgery was over, all patients were connected to an intravenous infusion pump for analgesia. Patients receiving hydromorphone were administered an initial dose of hydromorphone 0.02 mg/kg (2 ml) and a maintenance dose of hydromorphone 0.01 mg/kg/h and flurbiprofen axetil 5 mg/kg [2 ml/h and analgesia controlled by the parents of the pediatric patient (1 ml)]. Patients receiving esketamine were administered an initial dose of esketamine 0.3 mg/kg (2 ml) and a maintenance dose of esketamine 0.15 mg/kg/h and flurbiprofen axetil 5 mg/kg [2 ml/h and analgesia controlled by the parents of the pediatric patient (1 ml)]. Patients, parents, and investigators were blinded to analgesic drug allocation.

### Data collection and outcomes

Patients' demographic and clinical characteristics, including age, weight, grade of hypospadias, intraoperative blood loss, operative time, and awaking time were recorded. Postoperative pain was assessed using the Face, Legs, Activity, Cry and Consolability (FLACC) scale (8). The primary endpoint was the FLACC score and secondary endpoints included the Ramsay sedation score (9). FLACC and Ramsay sedation scores were recorded when leaving the PACU (0 h) and at postoperative 2, 6, 12, 24, 36, and 48 h. Postoperative complications, including scores on the Pediatric Anesthesia Emergence Delirium Scale (PAED) (10), incidence of hypotension, respiratory depression, nausea and vomiting and pruritus, and the time to first bowel movement after surgery, were recorded. Hypotension was diagnosed when systolic blood pressure was <20% of the baseline blood pressure. Respiratory depression was diagnosed when SpO_2_ was <92%. Urine samples (5 ml) were collected from drainage bags 24 h after postoperative urinary retention, and urinary tryptophan, 5-hydroxytryptamine, arginine, and substance P levels were detected by ELISA.

Postoperative pain on the ward was managed by an investigator from the Department of Anesthesiology. When FLACC scores were >4, an investigator administered a 2-ml bolus of rescue analgesia from the intravenous infusion pump. Patients who required >2 analgesic rescues were administered intravenous sufentanil 0.05 µg/kg every 10 min until the FLACC score was ≤4.

### Statistical analysis

**Figure 1:**
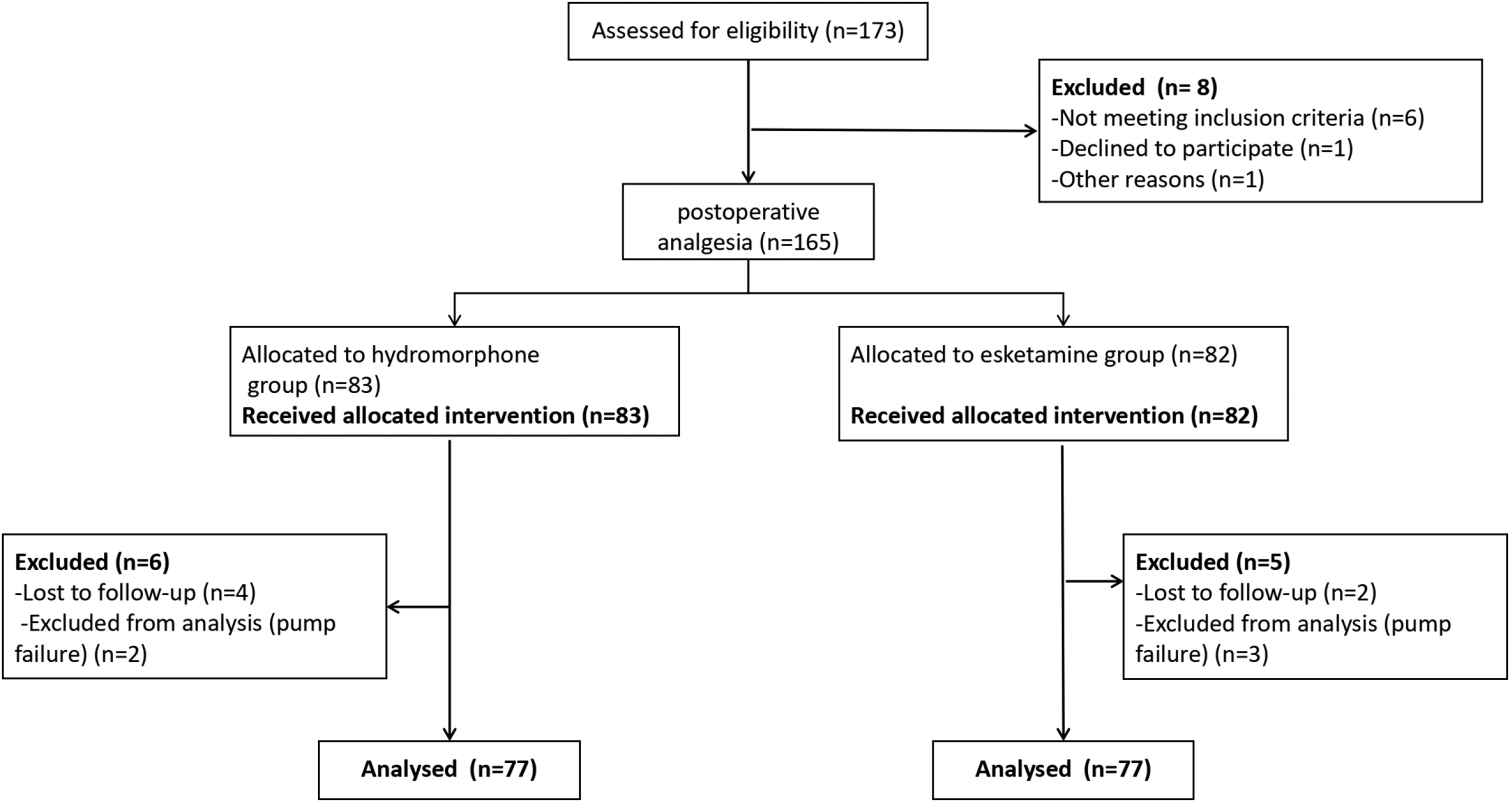
Study flow chart.

Sample size was calculated using the PASS 11.0 Sample Size software program Sample size was calculated based on a pilot study that found mean FLACC scores for patients in the H group or E group were 2.8 or 3.2, respectively, and the standard deviation was 0.8. Assuming *α *= 0.05 and *β *= 0.2, the sample size was calculated as 64 patients per group. Assuming 20% of patients may be lost to follow-up, the sample size was calculated as 77 patients per group (Figure 1).

Continuous variables are summarized as mean and standard deviation, and were compared using the *t-*test. Categorical variables are expressed as frequency (percentage), and were compared using the *χ*^2^ test. *P *< 0.05 was considered statistically significant.

## Results

Patients' demographic and clinical characteristics are summarized in Table 1. There were no significant differences in age, weight, grade of hypospadias, blood loss during operation, operative time, and awakening time from anesthesia between the H group and the E group.

**Table 1:** Patients’ demographic and clinical characteristics.

	H	E	*t*/*x*^2^	*P*
Age (years)	3.44 ± 0.27	3.43 ± 0.27	0.386	0.700
Weight (kg)	14.56 ± 0.87	14.58 ± 0.9	0.198	0.843
Grade of hypospadias: Glans Penis and Scrotal/Perineal	38/31/8	36/34/7	0.259	0.878
Intraoperative blood loss (ml)	34.66 ± 3.47	35.38 ± 3.02	1.364	0.175
Operative time (h)	2.53 ± 0.50	2.60 ± 0.49	0.809	0.420
Awaking time from anesthesia (min)	25.36 ± 3.35	25.45 ± 2.96	0.178	0.859

**Figure 2:**
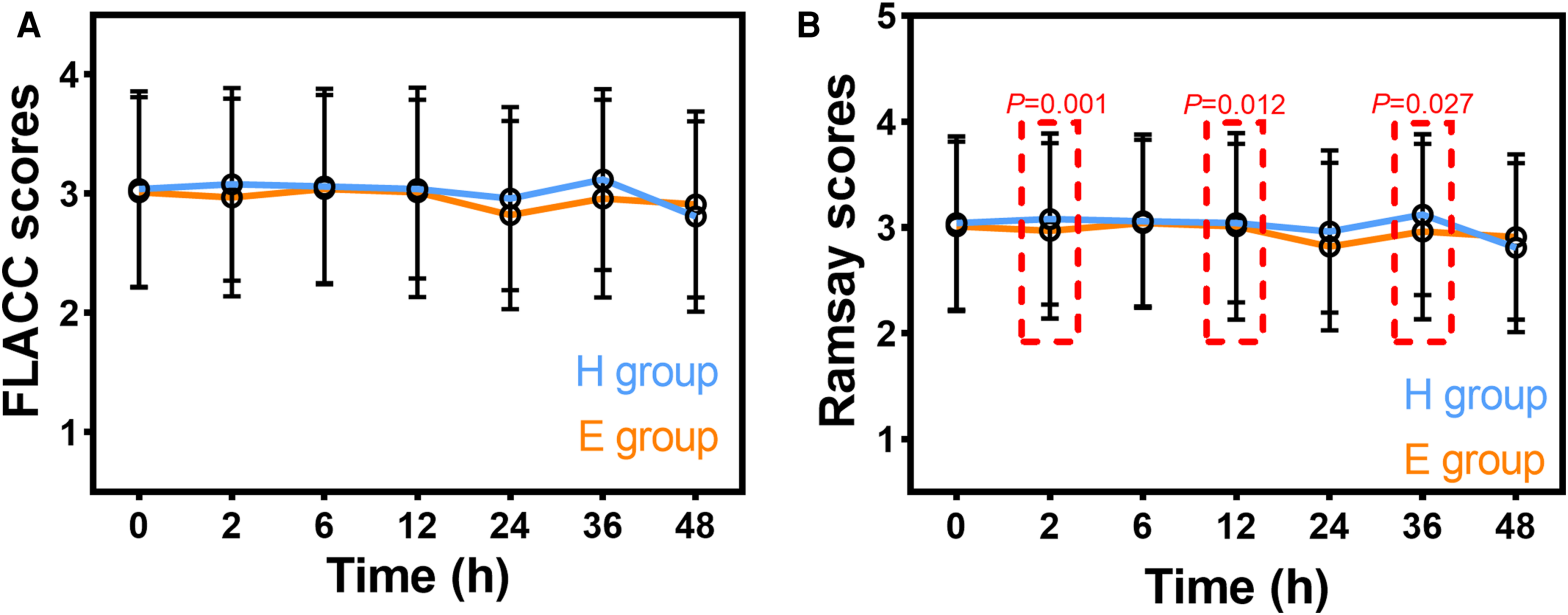
Postoperative FLACC scores (**A**) and Ramsay sedation scores (**B**) in patients receiving hydromorphone (H group) or esketamine (E group). FLACC: Face, Legs, Activity, Cry, Consolability scale. Data are mean ± standard deviation.

FLACC scores at postoperative 0, 2, 6, 12, 24, 36, and 48 h are summarized in Figure 2A. FLACC scores were all <4. There were no significant differences in FLACC scores at postoperative 0, 2, 6, 12, 24, 36, and 48 h in intra- and inter-group comparisons. Ramsay sedation scores at postoperative 0, 2, 6, 12, 24, 36, and 48 h are summarized in Figure 2B. There were no significant differences in Ramsay sedation scores at postoperative 0, 2, 6, 12, 24, 36, and 48 h in the intra-group comparisons in the H group or the E group. Ramsay sedation scores were significantly lower at postoperative 2, 12, and 36 h in the H group compared to the E group.

Postoperative complications are summarized in Table 2. There were no significant differences in the PAED scale or incidence of nausea and vomiting or pruritus between the H group and the E group. The incidence of hypotension and respiratory depression was significantly lower, and the time to first bowel movement was significantly shorter in the E group compared to the H group.

**Table 2:** Postoperative complications.

	H	E	*x*^2^/*t*	*P*
PAED scale (mean ± SD)	8.73 ± 1.03	8.40 ± 1.14	1.852	0.066
Hypotension [*n*(%)]	12 (15.6)	4 (5.2)	4.464	0.035
Respiratory depression [*n* (%)]	13 (16.9)	5 (6.5)	4.026	0.045
PONV [*n* (%)]	2 (2.6)	3 (3.9)	0.207	0.649
Pruritus [*n* (%)]	2 (2.6)	1 (1.3)	0.340	0.560
Time to first bowel movement (h, mean ± SD)	32.18 ± 1.57	29.06 ± 0.82	15.451	<0.001

PAED, Pediatric Anesthesia Emergence Delirium Scale; PONV, postoperative nausea and vomiting; SD, standard deviation.

**Figure 3:**
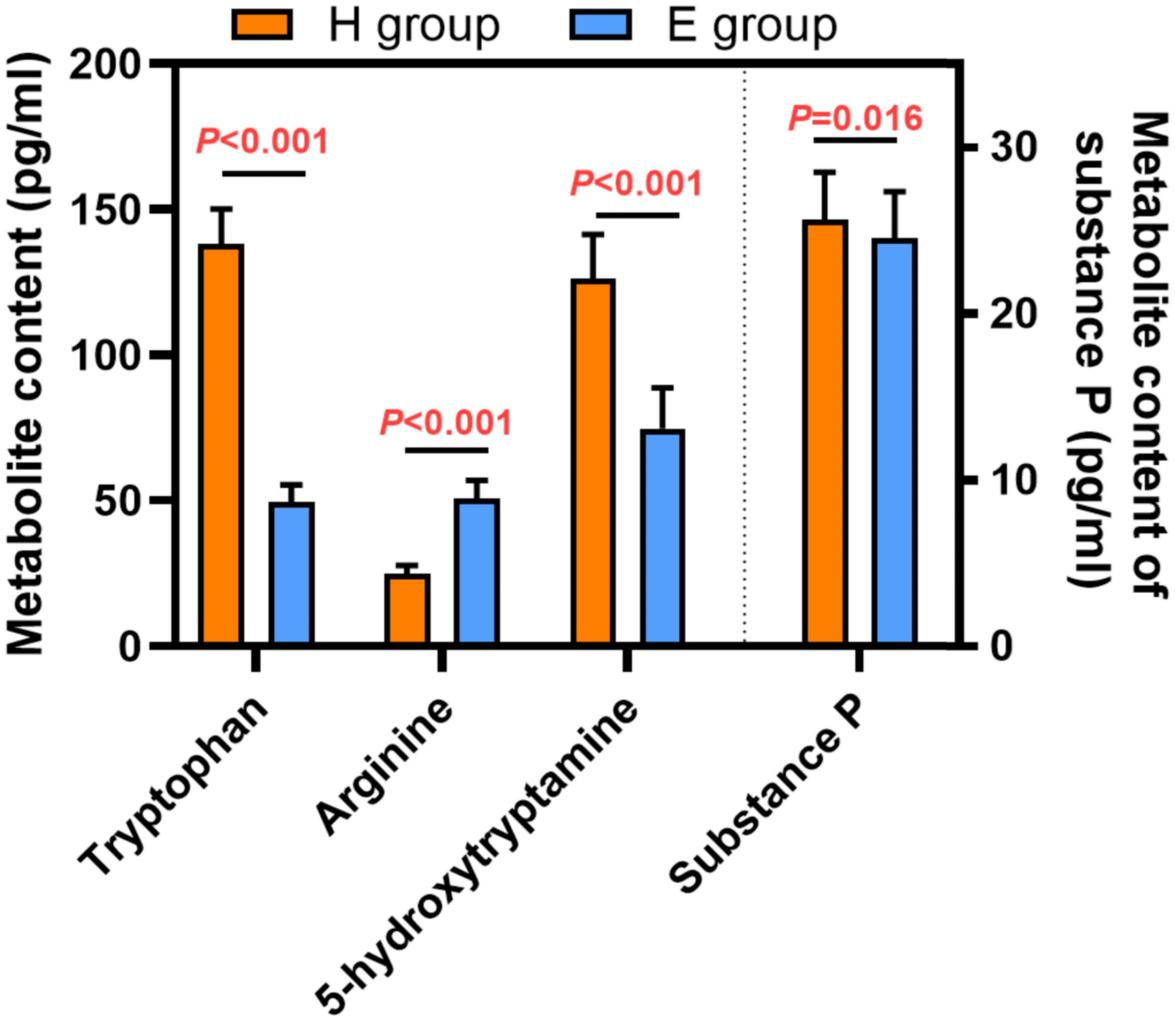
Urinary metabolites in patients receiving hydromorphone (H group) or esketamine (E group). Data are mean ± standard deviation.

Urinary metabolites are summarized in Figure 3. Urinary tryptophan, 5-hydroxytryptamine and substance P levels were significantly lower but arginine was significantly higher in the E group compared to the H group.

## Discussion

This study explored the safety and efficacy of the combination of continuous intravenous infusion of esketamine with sacral block for postoperative analgesia in pediatric patients undergoing surgery for hypospadias.

Ropivacaine is used for a single sacral block in the patient, and the analgesic effect is only maintained for 6 h. Continuous intravenous infusion of analgesic can provide postoperative analgesia for 48 h (11). Findings showed that esketamine or hydromorphone provided an efficient way to manage pain after surgical repair of hypospadias, esketamine had a stronger sedative effect than hydromorphone at postoperative 2, 12, and 36 h, and the incidence of postoperative complications appeared lower in patients who received esketamine compared to patients who received hydromorphone. Postoperative pain following surgical repair of hypospadias is severe and can be associated with disturbed sleep, which can result in changes in behavior and poor emotional well-being (12). The results of this study imply that the combination of continuous intravenous infusion of esketamine with sacral block can provide effective analgesia and sedation for this pediatric patient population.

Ketamine is an intravenous anesthetic with sedative, analgesic, and anesthetic effects (13). Esketamine is an isomer of ketamine with a similar mechanism of action. However, esketamine has stronger anesthetic and analgesic effects than ketamine, because esketamine has a much higher affinity for NMDA receptors than ketamine (14). Esketamine has a rapid onset, few side effects and an obvious analgesic effect, which allows broader clinical application than ketamine. Esketamine exerts hypnotic, sedative, and analgesic effects by interacting with the NMDA receptor, opioid receptor, M choline receptor, monoamine receptor, adenosine receptor, and other purine receptors (15). Evidence suggests esketamine is an effective pharmacological option for pain relief. Esketamine can be used as an adjuvant analgesic and reduce the consumption of opioid analgesics in patients undergoing spinal surgery or craniotomy (16, 17), and has utility in the routine treatment of refractory pain (18).

Hydromorphone is an opioid agonist that is often used for pediatric analgesia. Opioids provide effective analgesia, but can cause nausea and vomiting, constipation, urinary retention, and respiratory depression (19); therefore, their use in pediatric patients should be minimized (20). In the present study, there were no significant differences in the PAED scale or incidence of pruritus or nausea and vomiting between patients who received hydromorphone and patients who received esketamine. The incidence of hypotension and respiratory depression was significantly higher, and the time to first bowel movement was significantly longer, in patients who received hydromorphone compared to patients who received esketamine.

Urinary tryptophan, 5-hydroxytryptamine, arginine, and substance P levels were measured to investigate the physiological mechanisms underlying the lower incidence of several postoperative complications associated with esketamine compared to hydromorphone in our patient population. Urinary tryptophan, 5-hydroxytryptamine,, and substance P levels were significantly lower but arginine was significantly higher in patients who received esketamine compared to patients who received hydromorphone. Tryptophan is an essential amino acid that plays an important role in maintaining nitrogen balance, muscle mass, and body weight. Tryptophan participates in several metabolic pathways and is a precursor of 5-hydroxytryptamine (5-HT) (21, 22). As an emetic substance, 5-HT is expressed in the body after surgery and anesthesia. This implies that the effect of esketamine on reducing nausea and vomiting compared to hydromorphone may be related to the reduction of serotonin and its precursor. Arginine is metabolized to nitric oxide, and can reduce intestinal mucosal damage (23). In the present study, levels of urinary arginine were significantly higher in patients who received esketamine compared to patients who received hydromorphone, so the time to first bowel movement was significantly shorter in patients who received esketamine. Further studies are required to fully understand the mechanism of action of esketamine in pediatric patients.

This study was associated with several limitations. It was a single-center study that used only one dose of esketamine. A multi-center study evaluating the safety and efficacy of different doses of esketamine for postoperative analgesia in pediatric patients is warranted.

## Conclusions

The combination of continuous intravenous infusion of esketamine with sacral block provided safe and effective postoperative analgesia for pediatric patients undergoing surgery for hypospadias.

## Data Availability

The raw data supporting the conclusions of this article will be made available by the authors, without undue reservation.
